# Knowledge-for-data trade at the interface between precision medicine and person-centered care

**DOI:** 10.3325/cmj.2018.59.132

**Published:** 2018-06

**Authors:** Srećko Gajović

**Affiliations:** Croatian Institute for Brain Research, University of Zagreb School of Medicine, Zagreb, Croatia *srecko.gajovic@hiim.hr*

The interaction between the individual citizen and the health care system is a key element of the person-centered care. The two sides, the citizens and the system, the users and the provider, have a common interest in achieving health, but are in conflict over the control issues. In this article, I suggest that the equilibrium between the individuals and the health care system could be achieved through the exchange of patient’s personal data for the knowledge on how to be treated. This exchange has already been present occasionally, but has provided only additional benefits for the patients. With the onset of digital technologies and the introduction of the precision medicine, it could be a mode satisfying both sides. Such a trade presents high risks for the individual citizens, although promising them the benefits of up-to-date medical care through precision medicine.

## The challenge of person-centered care

Person-centered care is a health care concept in which individuals control their health. Consequently, citizens should be in the position to individually choose, monitor, and influence any activity related to their health and the health of their communities. It stands in contrast to medical paternalism, where medical professionals decide on medical interventions needed (1).

Person-centered care is a result of two mutually dependent processes. The first is the increasing recognition of human rights and respect of individual autonomy and privacy. This is codified by the Universal Declaration of Human Rights and the Declaration of Helsinki, and further developed through the principles of bioethics and medical ethics. In short, every individual should have the right to decide about his or her own body and mind, in particular in relation to health issues (2). The second process is the technological advancement of medicine. The extensive and complex medical records contain many different types of data (text, numbers, graphs, or images) for almost every individual. At the same time, different medical specialties produce separate sets of medical records, all of which are legally owned by the patient, who has the right and duty to take care of them.

The two processes already exist, but person-centered care still needs to be justified and discussed (3). The reason for this is an extremely complex health care system. To imagine a user-centered system is easy, just let us look at the libraries. One can enter the library, register, and choose the book according to his or her own preferences. Librarians are there to provide support and advice. If the book is boring or inadequate, it can be easily replaced. The interested individual could also move to another library offering different book collections.

The most obvious difference between libraries and the health care system is that a wrong therapy could be detrimental to the person and is connected with legal liabilities. Next, choosing the right medical procedure is knowledge- and technology-intensive process. This includes coordinated education of all medical professionals; planning, acquiring, and maintaining the necessary infrastructure; plus managing the system to provide care efficiently and openly to all in need. In addition, the health care system is very costly, and it represents an important part of the societal economic activities. A complex billing system is installed to charge individuals, insurers, public bodies, and taxpayers for the costs of the health service. Health economy accounts for more than 10% of gross domestic product of the most developed nations, predicted to increase in the near future to as much as 20%.

In conclusion, although the person-based care assumes that individuals should control their own body, mind, and health, the health care system's complexity, financial value, and societal importance are so high that the system remains in charge. The conflict between users and providers is tamed by the notions that their common goal is health and that the current health care system is organized around and for the patients. There is no doubt that citizens and health care system are partners in the process. Still, recognizing the conflict and dealing with it is essential for person-centered care implementation. To understand and solve this conflict we need to get insight into the distinctive perspectives of the two parties, and pinpoint the differences between them.

## The health care system perspective

The health care system perspective is oriented to get the system efficiently running, providing high-quality care for “reasonable” cost. The efficiency of the health care system depends on a complex list of demands, including the availability of care, new technologies, multiparty workflows, dynamic legal requirements, and reliable cost accounting (4). To meet some of these demands, digital technologies such as eHealth are increasingly used (5). eHealth provides managing solutions by advanced analytics of the integrated data. I believe that this description “providing managing solutions by advanced analytics of the integrated data” could characterize the core business of the digitally enhanced health care system. Integrated eHealth medical records help physicians to more easily access accumulated patients’ data and make these data available for the “advanced analytics,” which can provide reference values and process or even interpret data. On the basis of integrated and analyzed individual data, the informed medical teams are expected to provide exact individualized treatments, the notion represented in the concept of the precision medicine (6). The precision medicine enables the patients to get precisely the exact medical treatments they need, representing a key technology of eHealth.

Together with providing individualized solutions to the patients, the digital technologies are also crucial for the system itself. This includes managing the workflow by eAppointments, providing drugs through eRecepies, and keeping eRecords of the procedures. eHealth makes health care more efficient and less costly than it used to be.

eHealth functioning is data-dependent. To live up to its societal expectations, digitalized health care system is thirsty for data. Still, the data do not belong to the system but to the patients (7). This dependency on a large quantity of data owned outside the system creates a very weak spot from the health care perspective. Therefore, the health care system perspective in the context of person-centered care is characterized by a lack of the very foundation of its functioning – the patients’ data.

## The individual citizen’s perspective

The individual citizen’s perspective is rather different. There is no subset of citizens who are primarily patients. All individuals are concerned about health, be it their own health, their loved ones' health, or local and global community's health. This in particular includes healthy people, including “top performers,” as their continuous and successful activities depend on their health status (8).

The individual perspective in a search for health is opposite to the search for data. The individual citizens need the knowledge on how to interpret their personal data, combat the illness, and reach health. In contrast to health care system, which is thirsty for data, the persons seeking health are thirsty for knowledge. The exchange of knowledge for data comes natural. This exchange is not a new feature of digital society or a peculiarity of person-centered medicine. The same exchange occurred in the system based on medical paternalism. The patient would present their symptoms and get the knowledge on how to be cured. The data had little value (the anonymity needed to be preserved indeed), and the knowledge was a key asset of the physicians, giving them the dominant role. Although the exchange of knowledge-for-data occurred during medical intervention, this did not have the wide implications present today, when enormous quantities of personal medical data are generated on a continuous basis.

## The interaction of health, knowledge, and data

By the onset of the digital society, the value of health, knowledge, and data had increased immensely. The recently created Navigating Knowledge Landscapes Network has acknowledged the importance of knowledge in the digital society (9). Describing the individual search for health as navigation through the knowledge landscapes, they have recognized and highlighted the key target of the search – knowledge. The individual quest for health represents a search for health-related knowledge (10).

This search for knowledge extends greatly in the digital society. The health advice is not asked for only in the physician’s practice, or by talking to the family, relatives, or friends. The search for knowledge in the digital environment includes browsing through web pages, digital repositories, and social networks. Knowledge is global and always available. Digital environment empowers the patient by providing at least partial independence from the health care system and offering alternative knowledge resources. This improves the position of individuals as health care users, but it also exposes them to previously unknown risks (11).

The digital society also improves the health care system, which is positioned at the intersection of health, knowledge, and data, all three being the candidates for the key currencies of the digital economy. Being healthy represents the ultimate wealth, as it cannot be acquired through instant shopping and as it requires a long-term careful investment strategy. The knowledge represents a key resource of innovations for the current knowledge-based economy, while the data are the basis of the new system of “dataism,” giving more power to data ownership than to capital ownership, hence replacing “capitalism” (12).

The described exchange of knowledge for data gets a new dimension in the current digital setting. The available technologies enable combining of information obtained by genetic and other -omics technologies with body sensors, adding the real-time monitoring aspect to data collection (13). This is augmented by increasing data processing power and emergence of artificial intelligence expected to interpret the collected data (14). The purpose of data processing would be not only to precisely cure the individual, but to prevent illness and maintain the health of every citizen. Having every (or at least many) citizens involved would provide the desired benefit through big data analysis (15). Hence, many citizens would be willing to tick the box “I accept,” if the core of the promise was health. This makes the knowledge-for-data trade so important and likely to be the base of the future health care.

As the data value would inevitably rise, the knowledge-for-data trade can evolve as a key site where societal forces can “buy” or “steal” personal data. Citizens might be instructed to voluntarily give up all their personal data to be included into “big data” analysis improving the health of community, nation, or society. Giving up personal data to the benefit of society could be considered as a rather positive notion (16). Therefore, the knowledge-for-data trade in the health arena could be a key element for the overall loss of individual private data ownership. The depreciation of data ownership and appreciation of the knowledge provided (in particular through precision medicine) could provide the key entry site for private data to be imported to the system. This would not only be a demise of person-centered care, but a creation of new “digital paternalism” (eg, advanced analytics of integrated data would indicate “precisely” what this individual should be – history teacher, policeman in a particular suburb, or cab driver…). The insight in the personal data could provide the decision power to the governing bodies, which if concentrated in the hands of the few, could undermine democracies and lead to digital dictatorships.

On the other hand, keeping individuals aware of their personal data value and protecting the individual data ownership legally (eg, through General Data Protection Regulation recently introduced in the European Union) would empower individuals (17). Subsequently, the knowledge-for-data trade could favor the individual citizens. In this way, the knowledge-for-data trade could be a major asset in establishing the person-centered care, centered around data owners. Appreciating the value of private data could be a major tool to protect individual rights and allow for the survival of democracy in the digital society.

In conclusion, the knowledge-for-data trade represents a critical feature of the interaction between individual citizens and health care system. This trade is crucial for the establishment of both precision medicine and person-centered care. The result of the trade could lead in two opposite directions: loss of private data ownership, medical paternalism, and digital dictatorships or empowering the private data ownership, person-centered care, and digital democracies.
